# A categorical approach to neurodynamical modelling of musical tonality

**DOI:** 10.1186/1471-2202-16-S1-P101

**Published:** 2015-12-18

**Authors:** Michal Hadrava, Jaroslav Hlinka

**Affiliations:** 1 Department of Cybernetics, Faculty of Electrical Engineering, Czech Technical University in Prague, Prague, 166 27, Czech Republic; 2 Department of Nonlinear Dynamics and Complex Systems, Institute of Computer Science, The Czech Academy of Sciences, Prague, 182 07, Czech Republic

## 

Music gives rise to some of the strongest emotional experiences in our lives. Both musicological and music-psychological evidence converge on the theory that music achieves this by a sophisticated play with our expectancies [1]. The extent to which a pitch/chord is expected in the context of a musical sequence is closely related to its perceived "stability" and also to how much it apparently "attracts" other pitches/chords in the sequence (see [2] and the references thereof). The phenomenon wherein more stable pitches attract the less stable ones is called tonality.

Over the eons, virtually every musical culture has created its own set of musical scales. Generally, each scale imposes a hierarchy of stability on all pitches sounded in its context [3]. We call such a hierarchy a tonal hierarchy. In [4] a formula for computing tonal stability of arbitrary pitch in a given context was proposed based on a network of neural oscillators. Even though high correlations with tonal hierarchies obtained experimentally were achieved by tuning the single parameter of the formula, there were some notable discrepancies between predictions of the formula and the psychological data. These might be explained by drastic approximations made on the way from the complete neurodynamical model to the formula which were necessitated by the complexity of the model. For instance, all interactions within the network were neglected. Nevertheless, this line of research clearly demonstrates the potential of neurodynamics to serve as a basis for derivation of a general theory of tonality applicable even to contemporary art music which is our main interest. Indeed, the neurodynamical model from [4] makes no assumptions whatsoever regarding its inputs. What is currently missing is a model of a suitable form so that such a theory can be derived from it without drastic approximations. We aim to fill this gap.

It has become a standard in neuroscience community to approach neurodynamics from a dynamical systems perspective. Unfortunately, none of the approaches to analytical treatment of oscillatory dynamical systems we have reviewed proved suitable for our purposes. Consequently, inspired by the so-called "memory evolutive neural systems" [5], we are currently developing a neurodynamical model based on category theory. More specifically, we model the neural rhythms present in the auditory system during stimulation with a tone/chord as a category with objects representing the rhythms and morphisms representing the resonant interactions among them. Response to a sequence of stimuli is modelled using partial functors between such categories. The preliminary results are quite promising: from a very simple model we managed to derive a formula which predicts the ordering of pitches according to tonal stability within a major scale almost perfectly. Moreover, due to its nature, our model yields an almost mechanistic explanation of tonality. This, in our opinion, is as important a feature of any model as a good fit to data.
